# Prolongation of Cardiac Allograft Survival by Endometrial Regenerative Cells: Focusing on B‐Cell Responses

**DOI:** 10.5966/sctm.2016-0206

**Published:** 2016-10-26

**Authors:** Xiaoxi Xu, Xiaochun Li, Xiangying Gu, Bai Zhang, Weijun Tian, Hongqiu Han, Peng Sun, Caigan Du, Hao Wang

**Affiliations:** ^1^ Department of General Surgery, Tianjin Medical University General Hospital, Tianjin, People’s Republic of China; ^2^ Tianjin General Surgery Institute, Tianjin Medical University General Hospital, Tianjin, People’s Republic of China; ^3^ Department of Cardiology, Tianjin Medical University General Hospital, Tianjin, People’s Republic of China; ^4^ Department of Gynecology and Obstetrics, Tianjin Medical University General Hospital, Tianjin, People’s Republic of China; ^5^ Department of General Surgery, Affiliated Hospital of Weifang Medical University, Shandong, People’s Republic of China; ^6^ Department of Urologic Sciences, University of British Columbia, Vancouver, British Columbia, Canada

**Keywords:** Heart transplantation, Endometrial regenerative cells, B lymphocytes, Antibody‐mediated rejection, Immunosuppression, Mice

## Abstract

Endometrial regenerative cells (ERCs) have been recently evaluated as an attractive candidate source for emerging stem cell therapies in immunosuppression, but their role in immunoregulation is not fully understood. The present study was designed to investigate their effects, especially on B‐cell responses in heart transplantation. In this study, ERCs were noninvasively obtained from menstrual blood. Heart transplantation was performed between C57BL/6 (H‐2^b^) donor mice and BALB/c (H‐2^d^) recipients. B‐cell activation and antibody levels were determined using fluorescence‐activated cell sorting, enzyme‐linked immunosorbent assay and ELISpot. In this study, we demonstrated that ERCs negatively regulated B‐cell maturation and activation in vitro without affecting their viability. ERC treatment prolonged cardiac allograft survival in mice, which was correlated with a decrease in IgM and IgG deposition and circulating antidonor antibodies, as well as with reduction in frequencies of antidonor antibody‐secreting CD19^+^ B cells. In addition, upon ex vivo stimulation, B cells from ERC‐treated heart transplant recipients had impaired proliferation capacity and produced less IgM and IgG antibody. Moreover, ERC treatment of mice receiving ovalbumin (OVA)‐aluminum hydroxide vaccine resulted in significant lower numbers of anti‐OVA IgG antibody‐secreting splenic B cells and lower anti‐OVA antibody titres. Our results indicate that therapeutic effects of ERCs may be attributed at least in part by their B‐cell suppression and humoral response inhibition, suggesting the potential use of ERCs for attenuating antibody‐mediated allograft rejection. Stem Cells Translational Medicine *2017;6:778–787*

**Significance Statement**

The alloantibody‐associated episodes of acute and chronic allograft rejection are still prevalent in the clinic. This study demonstrates that endometrial regenerative cells (ERCs), a novel source of adult mesenchymal stem cells noninvasively obtained from menstrual blood, inhibit B‐cell activation and differentiation with reduced antibody production in a mouse cardiac transplant model. The unique features of the ease of collection, relatively unlimited source, immunomodulatory effect, and hypoimmunogenicity could make ERCs an attractive candidate source for stem cell therapies for the prevention and/or treatment of acute and chronic humoral rejection following transplantation.

## Introduction

Short‐term (1‐year) allograft survival rates have been improved to 88%–95% with current effective immunosuppressive therapies [1]. However, the alloantibody‐associated episodes of acute and chronic allograft rejection are still prevalent in clinic [2, 3, 4, 5, 6, 7]. Although there are therapeutic strategies available for the prevention of the antibody‐mediated rejection by either depleting the resting B cells [8, 9, 10] or removing the alloantibody from circulation [11, 12, 13, 14], none of them is functionally effective in the inhibition of B‐cell activation and/or differentiation.

Mesenchymal stem cells (MSCs) have been recently tested as an emerging immunosuppressive therapy for prevention of transplant rejection; they inhibit the responses of dendritic cells (DCs) [15], T cells [16], and B cells [17]. Recent studies have found that MSCs directly regulate B‐cell activation and maturation [18, 19]. However, some limitations of the use of MSCs from the current sources (i.e., bone marrow, adipose tissue, and umbilical cord) are identified, such as the possibility of tumorigenesis [20], promoting tumor progression [21], and increasing the metastatic potency of cancer cells [22]. Furthermore, the invasive obtaining procedure, less availability, and limited proliferation capacity should be taken into account when considering the application of MSCs as a cell therapy.

Endometrial regenerative cells (ERCs) can be derived from menstrual blood and recently receives much attention as a novel source of adult MSCs for their phenotypic characteristics as well as the immunomodulatory properties [23, 24]. Apart from the ease of abstraction and the abundant source, ERCs possess following advantages: (1) capability of expanding up to 68 doublings while maintaining karyotypic normality and differentiation ability; (2) higher proliferative rate with doubling occurring every 19.4 hours; (3) production of high levels of growth factors and matrix metalloprotease, which favors tissue repair; and (4) greater pluripotency of differentiating into 9 lineages [23, 25]. We and others have reported that ERCs are capable of preventing critical limb ischemia [23], reducing myocardial infarction [26], and attenuating experimental colitis [27]. However, their immunosuppressive activities against B‐cell functions or B‐cell‐mediated transplant rejection have yet to be investigated. Thus, the present study was designed to determine the immune‐suppressive effects of ERCs on B cells, particularly in the setting of cardiac allograft rejection.

## Materials and Methods

### Animals

C57BL/6 (H‐2^b^), BALB/c (H‐2^d^), and C3H (H‐2^k^) mice (Aoyide Co., Tianjin, China) were used in the present study. All experiments were conducted in accordance with the protocols approved by the Animal Care and Use Committee of Tianjin Medical University (Tianjin, China) according to the Chinese Council on Animal Care guidelines.

### Cell Preparations

ERCs were prepared following the protocol described previously [26, 28]. In brief, after informed consent was obtained, menstrual blood was collected from six healthy women (20–30 years old) on the first day of menstruation using a menstrual cup. Mononuclear cells were fractionated by Ficoll‐Paque density gradient centrifugation. The samples were suspended in Dulbecco’s modified Eagle’s medium (DMEM) high glucose supplemented with 10% fetal bovine serum (FBS) and 1% penicillin/streptomycin, and they were split into two 10‐cm dishes. The purity of adherent ERCs was more than 80%, and the cell number was approximately 1 × 10^7^ cells in the initial cultures [26]. Culture medium was changed the next day. The cells were then subcultured and passaged twice per week. ERCs adhered to plastic dishes in cultures and showed spindle shape morphology. Typical cell surface markers of ERCs were analyzed by flow cytometry as previously described [25]. Third‐ and fourth‐passage cells were used for treatment.

Splenic CD19^+^ B cells were isolated from the spleens of BALB/c mice (naïve and/or heterotopic cardiac allograft recipients) by positive selection using magnetic activated cell sorting beads (Miltenyi Biotec, Auburn, CA, http://www.miltenyibiotec.com) according to the manufacturers’ protocol.

### Treatment With ERCs

ERC‐treatment of both allograft recipients and ovalbumin (OVA)‐immunized mice was performed by i.v. injection of ERCs (1 × 10^6^ cells per mouse) 24 hours after cardiac transplantation or immunization. Mice receiving the same amount of DMEM/FBS medium were vehicle controls.

### In Vitro B‐Cell Proliferation Assays

Splenic CD19^+^ BALB/c B cells were stimulated with lipopolysaccharide (LPS; Sigma‐Aldrich, St. Louis, MO, https://www.sigmaaldrich.com) in the absence or presence of irradiated ERCs. ^3^H‐Thymidine incorporation (GE Healthcare Bi‐Sciences, Pittsburgh, PA, http://www.gelifesciences.com) was measured as a proliferation index of B cells as previously described [29].

### Determination of Cell Viability and Apoptosis

The cell viability was determined using trypan blue stain exclusion assay (Thermo Fisher Scientific, Waltham, MA, https://www.thermofisher.com), and apoptotic cells were identified using flow cytometry with Annexin V fluorescein isothiocyanate (FITC) and 7‐aminoactinomycin D (7‐AAD) (BD Biosciences, San Jose, CA, http://www.bdbiosciences.com) staining as described previously [30].

### B‐Cell Surface Marker Expression

Coexpression of B‐cell surface markers was characterized using flow cytometry with fluorescent‐conjugated monoclonal antibodies specific for B220, CD80, CD83, and CD86 (eBioscience, San Diego, CA, http://www.ebioscience.com) following manufacturer’s instructions.

### Enzyme‐Linked Immunosorbent Assay

The levels of antibodies in cell culture supernatants or serum samples were determined using enzyme‐linked immunosorbent assay (ELISA). IgM or IgG levels in the supernatants of in vitro‐stimulated CD19^+^ B cells or mouse sera were measured using Mouse IgM or IgG ELISA Quantitation Kits, respectively (Bethyl Laboratories, Montgomery, TX, https://www.bethyl.com) according to the manufacturer’s recommended protocol.

### Heterotopic Cardiac Transplantation

Intra‐abdominal heterotopic cardiac transplantation from C57BL/6 donors to BALB/c recipients was performed as previously described [31, 32]. Heartbeat of the grafts was evaluated as a marker of survival daily by abdominal palpation, and graft rejection was indicated by barely detectable cardiac impulses [31, 32].

### Graft Histology and Immunohistochemistry

Heart tissue samples were collected at postoperative day (POD) 8 or at rejection endpoint as appropriate. Sections were prepared routinely in the laboratory for histology and immunohistochemistry [33, 34, 35]. Intragraft IgM/IgG deposition was quantified as previously described [32]. Negative controls were performed by omitting the primary antibodies.

### Circulating Donor‐Specific Antibody Production

Circulating antidonor (C57BL/6) IgM and IgG antibodies were evaluated in various recipient (BALB/c) sera using flow cytometry following incubation with allogeneic donor splenocytes as described previously [36]. In brief, serum samples were diluted with phosphate‐buffered saline (neat, 1:15 and 1:30), and their donor‐specific IgM or IgG antibody bindings were detected using flow cytometry with FITC‐conjugated goat F(ab’)_2_ anti‐mouse IgM (μ chain‐specific) or anti‐mouse IgG (H+L chain‐specific; Abcam, Cambridge, MA, http://www.abcam.com).

### Ovalbumin‐Aluminum Hydroxide Immunization

Mice were immunized with an ovalbumin‐aluminum hydroxide (OVA‐Alum) suspension on day 0 (100 μg OVA/100 μl saline + 100 μl Alum; Sigma‐Aldrich), followed by a boosting injection of OVA alone (100 μg OVA/100 μl saline) on day 7, in the absence of transplantation. Blood samples were collected before vaccine as a baseline and at days 7, 14, 21, 28 after the first OVA priming immunization.

### Detection of Antibody‐Secreting Cells by B‐Cell ELISpot

B‐cell ELISpot was used to determine the antibody‐secreting cells. In brief, multiscreen 96‐well flat‐bottom plates (EMD Millipore, Billerica, MA, http://www.emdmillipore.com) were coated with either (a) sonicated splenocytes isolated from either donor C57BL/6, third‐party C3H or syngeneic BALB/c mice or (b) OVA protein. Viable splenic CD19^+^ B cells isolated from allograft recipients, OVA‐immunized, or naïve mice as appropriate were then added to the plates. Goat anti‐mouse IgG‐Fc biotin conjugate (Sigma‐Aldrich) were used to detect the presence of antibody‐secreting cells (ASCs) [37].

### Statistical Analysis

Allograft survival data were presented as mean survival time (MST) and were analyzed using a log‐rank test. One‐way analysis of variance (ANOVA) and a two‐tailed, paired *t* test were used to analyze differences between experimental groups. Differences with *p* ≤ .05 were considered significant.

## Results

### ERC Treatment Inhibits the Proliferation of LPS‐Stimulated B Cells

**Figure 1:**
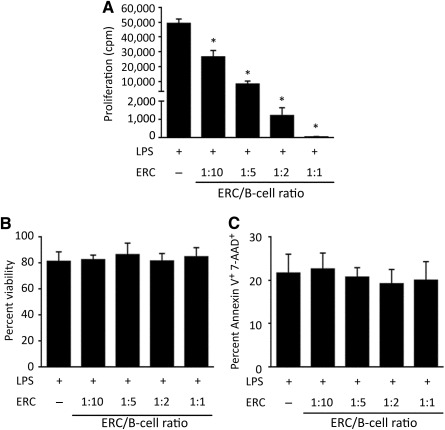
ERCs inhibit the proliferation of B cells without affecting their viability. Pure BALB/c CD19^+^ B cells (10^5^ per well) were stimulated with 2 μg/ml LPS and cultured alone or with ERCs at 1:20, 1:10, 1:5, 1:2, and 1:1 ratios of ERCs to B cells for 48 hours. **(A)**: To measure the proliferation of ERC‐treated B cells, 1 μCi of ^3^H‐thymidine was added. Eighteen hours thereafter, cells were harvested and ^3^H‐thymidine incorporation was measured. ∗, *p* < .001 vs. the proliferation of B cells without ERC treatment. **(B, C):** To evaluate the viability of ERC‐treated B cells, cells were harvested and cell death was determined by **(B)** trypan blue exclusion and light microscopy and **(C)** flow cytometry after staining with Annexin V FITC and 7‐AAD. **(A–C):** Graphs represent mean ± SE of triplicate samples. *p* value was determined by one‐way analysis of variance. Data shown are representative of three separate experiments performed. Abbreviations: 7‐AAD, 7‐aminoactinomycin D; cpm, count(s) per minute; ERC, endometrial regenerative cell; LPS, lipopolysaccharide; FITC, fluorescein isothiocyanate.

The effect of ERCs on the polyclonal expansion of B lymphocytes was first tested in LPS‐stimulated B‐cell cultures at 1:20, 1:10, 1:5, 1:2, and 1:1 ratios of ERCs to B cells. As shown in Figure 1A, exposure of B cells to ERCs induced a dose‐dependent suppression of B‐cell proliferation. Treatment of B cells at the ERC/B‐cell ratio of 1:20 had no inhibitory effect (data not shown), but the 1:10 ratio of ERCs to B cells caused significant inhibition (*p* < .001). Meanwhile, the highest ERC/B‐cell ratio of 1:1 completely inhibited B‐cell proliferation (*p* < .001; Fig. 1A).

To exclude the possibility that decreased ^3^H‐thymidine incorporation was caused by ERC‐induced B‐cell death, the cell death in these B‐cell cultures was examined using both trypan blue exclusion and flow cytometry after staining with Annexin V and 7‐AAD. Despite increasing ERC/B‐cell ratios, cell viability remained high and the degree of apoptosis was low, indicating that the observed decrease in B‐cell proliferation was not caused by ERC‐induced cell death (Fig. 1B, 1C).

### ERCs Inhibit B‐Cell Maturation/Costimulatory Marker Surface Expression

**Figure 2:**
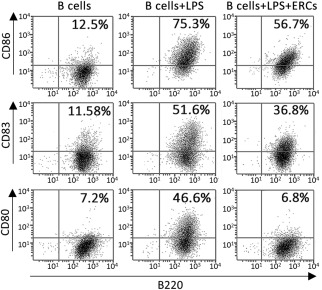
Differential inhibition of B‐cell maturation/costimulatory marker surface expression after treatment with ERCs. Pure BALB/c CD19^+^ B cells (2 × 10^6^ per well) were stimulated with 2 μg/ml LPS in the absence or presence of ERCs at 1:5 ratio of ERCs to B cells. After 72 hours of culture, cells were harvested and stained with fluorescently labeled anti‐B220 and anti‐CD80, anti‐B220 and anti‐CD83, or anti‐B220 and anti‐CD86. Surface coexpression of B220, CD80, CD83, and CD86 was detected by four‐color flow cytometry. Data shown represent three separate experiments, with similar effects observed in each. Abbreviations: ERCs, endometrial regenerative cells; LPS, lipopolysaccharide.

To test the effect of ERCs on B‐cell differentiation/maturation, we compared the surface expression of CD80, CD83, and CD86 on LPS‐stimulated B cells in the absence or presence of ERCs. As shown in Figure 2, LPS stimulation dramatically increased surface expression of CD80, CD83, and CD86 to 46.6, 51.6, and 75.3% in these B‐cell cultures, respectively. In the presence of ERCs, the surface expression of CD80 was reduced by 85.4%, CD83 by 28.7%, and CD86 by 24.7%. In particular, CD80 surface expression on ERC‐treated B cells was comparable with the baseline expression seen on unstimulated B cells (Fig. 2).

### ERCs Mediate the Inhibition of IgM and IgG Production

**Figure 3:**
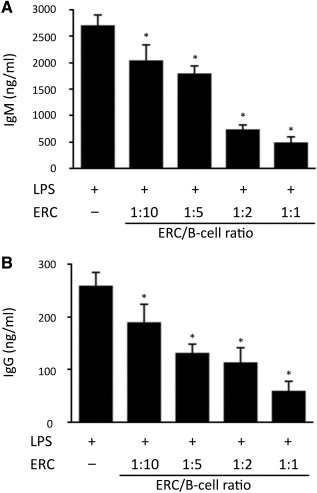
ERCs mediate inhibition of IgM and IgG production. Pure BALB/c CD19^+^ B cells (5 × 10^5^ per well) were stimulated with 2 μg/ml LPS with or without ERCs at 1:10, 1:5, 1:2, and 1:1 ratios of ERCs to B cells. After 6 days of culture, supernatants were harvested and **(A)** IgM or **(B)** IgG production was measured by enzyme‐linked immunosorbent assay. Graphs represent mean ± SE of triplicate wells. *p* value was determined by one‐way analysis of variance. Data shown are representative of three separate experiments. **(A):** ∗, *p* < .001 vs. the IgM level in the supernatants of B cells without ERC‐treatment. **(B):** ∗, *p* < .001 vs. the IgG level in the supernatants of B cells without ERC‐treatment. Abbreviations: ERC, endometrial regenerative cell; LPS, lipopolysaccharide.

To further confirm the inhibitory effect of ERCs on B cells, the IgM and IgG antibody levels in the supernatants of these B‐cell cultures were quantitated by using ELISA. As shown in Figure 3, ERCs significantly decreased both IgM and IgG production at a 1:10 ratio of ERCs to B cells (*p* < .001), and increasing ERC numbers further decreased IgM production. Similarly, despite low IgG production upon stimulation, ERCs induced a dose‐dependent trend in IgG suppression. The maximum suppressive effect on IgM and IgG production was observed at the 1:1 ratio of ERCs to B cells (*p* < .001; Fig. 3A, 3B).

### Treatment With ERCs Significantly Prolongs Murine Cardiac Allograft Survival

**Figure 4:**
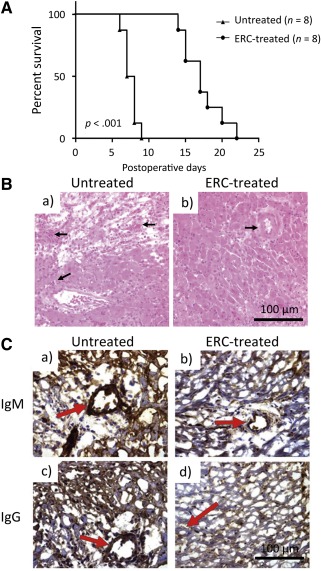
ERCs attenuate murine cardiac allograft rejection. **(A):** Prolonged murine cardiac allograft survival of ERC‐treated recipients. C57BL/6 (H‐2^b^) hearts were heterotopically transplanted to BALB/c (H‐2^d^) mice. In treated groups, recipients were injected i.v. with ERCs (1 × 10^6^ cells per mouse) 24 hours after cardiac transplantation. Beating of the grafted heart was monitored daily by direct abdominal palpation. End‐point for each animal represents cessation of cardiac impulses (*n* = 8). *p* value was determined by log‐rank survival test (∗, *p* < .001, survival proportions of recipients treated with ERCs vs. survival proportions of recipients without treatment). **(B):** Histology of cardiac allografts recipients. On postoperative day 8, the rejection time‐point for the untreated group, grafts were harvested and evaluated by H&E staining of paraffin sections (*n* = 8). **(Ba):** Cardiac grafts from untreated transplant recipients. **(Bb):** Grafts from ERC‐treated recipients. Arrows indicate intravascular and/or interstitial changes in heart grafts. **(C):** Intragraft IgM and IgG deposition in cardiac transplant recipients. Antibody deposition in grafted hearts at the time of sacrifice was observed by immunohistochemistry specific for either IgM or IgG (*n* = 8). **(Ca):** Cardiac grafts from untreated transplant recipients. **(Cb):** Grafts from ERC‐treated recipients. Arrows indicate IgM deposition around vessels. **(Cc):** Grafts from untreated transplant recipients. **(Cd):** Grafts from ERC‐treated recipients. Arrows indicate IgG deposition around vessels. Scale bars = 100 μm. Abbreviation: ERC, endometrial regenerative cell.

Our in vitro data suggested that ERCs mediated suppression of B‐cell activation in response to polyclonal LPS stimulus. To address if ERCs had similar actions in vivo, we assessed the therapeutic effect of ERCs on cardiac allograft survival of C57BL/6 (H‐2^b^) donors in BALB/c (H‐2^d^) recipients. As shown in Figure 4, the mean allograft survival time in ERC‐treated recipients was approximately two times longer than that in untreated recipients (*p* < .001; Fig. 4A). Microscopic examinations of heart allografts from untreated BALB/c recipients showed a typical feature of acute vascular rejection (AVR), characterized by predominant vasculitis (Fig. 4B), thrombosis, interstitial hemorrhage, and cell infiltration on POD8. In contrast, ERC treatment markedly attenuated graft pathological changes at the same day (Fig. 4B). These results demonstrate that ERC treatment is able to significantly prolong murine allograft survival, which is correlated with its attenuation of AVR.

### ERCs Significantly Decrease Intragraft IgM and IgG Deposition in Cardiac Transplants

To confirm if the therapeutic benefit of ERCs to cardiac allograft survival was attributed at least in part to their suppression of B‐cell response in vivo, the association of ERC‐mediated B‐cell suppression with the prolongation of allograft survival was investigated. As shown in Figure 4C, antibody deposition in grafted hearts on POD8 was observed by immunohistochemistry specific for either IgM or IgG. Whereas cardiac grafts from untreated transplant recipients exhibited a high density of IgM and IgG deposition around vessels (Fig. 4C) and in the interstitial tissue, the grafts from ERC‐treated recipients demonstrated only mild IgM and IgG deposition around vessels (Fig. 4C) and interstitial tissue.

### ERCs Impair the Proliferative Ability of B Cells Isolated From Cardiac Transplant Recipients

**Figure 5:**
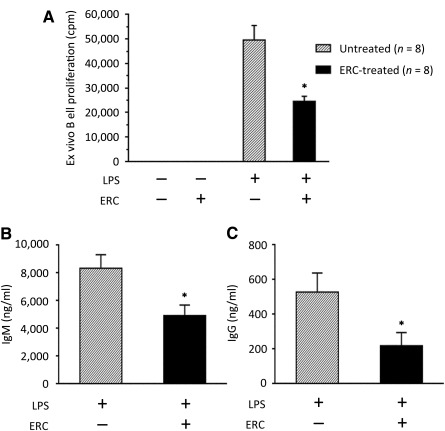
Ex vivo proliferation and IgM and IgG production of ERC‐treated recipient B cells after cardiac allografting. C57BL/6 (H‐2^b^) hearts were heterotopically transplanted to BALB/c (H‐2^d^) mice. In treated groups, recipients were injected i.v. with ERCs (1 × 10^6^ cells per mouse) 24 hours after cardiac transplantation. On postoperative day 8, the rejection time point for the untreated group, mice were sacrificed and recipient splenocytes were harvested (*n* = 8). **(A):** Purified recipient B cells (10^5^ per well) were stimulated in culture with 2 μg/ml LPS. After 48 hours of culture, 1 μCi of ^3^H‐thymidine was added. Eighteen hours thereafter, cells were harvested and ^3^H‐thymidine incorporation was measured. ∗, *p* < .001 vs. the proliferation of B cells from LPS‐stimulated, untreated recipients. **(B, C):** Purified recipient B cells (5 × 10^5^ per well) were stimulated in culture with 2 μg/ml LPS. After 6 days of culture, supernatants were harvested and **(B)** IgM or **(C)** IgG production was measured by enzyme‐linked immunosorbent assay. ∗, *p* < .001 vs. the levels of **(B)** IgM or **(C)** IgG antibody in the supernatants of B cells from untreated recipients. **(A–C):** Graphs represent mean ± SE of triplicate wells. *p* value was determined by a two‐tailed, paired *t* test. Data shown represent three sets of transplants and subsequently three separate ex vivo experiments. Abbreviations: cpm, count(s) per minute; ERC, endometrial regenerative cell; LPS, lipopolysaccharide.

To further test the association of ERC‐mediated B‐cell suppression with prolonged allograft survival (Fig. 4A), we directly evaluated the proliferation of B cells isolated from the recipients. Of note, after purification, splenic B‐cell numbers were not markedly different between ERC‐treated and untreated groups, with splenic B‐cell counts after purification ranging from 30 to 40 million per spleen (data not shown). We found that upon ex vivo LPS stimulation, B cells from ERC‐treated recipients had significantly lower proliferative capacity as compared with B cells from untreated recipients (Fig. 5A; *p* < .001).

### ERCs Decrease Ex Vivo IgM and IgG Production by B Cells Isolated From Cardiac Transplant Recipients

To further confirm the role of ERC‐mediated B‐cell suppression in the observed prolongation of allograft survival (Fig. 4A), we also compared the antibody producing capabilities of B cells purified from cardiac transplant recipients. As shown in Figure 5, upon ex vivo polyclonal LPS stimulation, B cells from ERC‐treated transplant recipients could produce only approximately half as much IgM and IgG in comparison with B cells from untreated recipients (*p* < .001 for IgM and IgG; Fig. 5B, 5C). Taken together, these data indicate that ERC treatment may have impaired B‐cell activation and antibody production in response to a fully major histocompatibility complex (MHC)‐mismatched cardiac allograft.

### ERCs Inhibit Antidonor Antibody Levels and Reduce the Frequency of Antidonor Antibody‐Secreting B Cells in Cardiac Allograft Recipients

**Figure 6:**
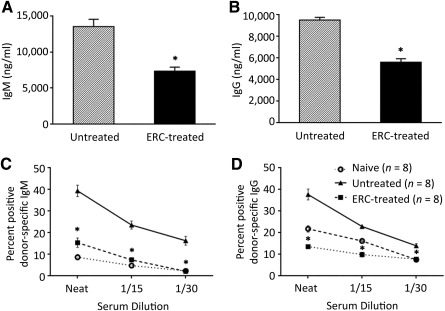
ERCs inhibit anti‐donor IgM and IgG levels in cardiac allograft recipients. **(A, B):** Sera from ERC‐treated and untreated heterotopic cardiac transplant recipients was harvested on postoperative day 8 (*n* = 8). Total **(A)** IgM or **(B)** IgG levels were measured by enzyme‐linked immunosorbent assay. Graphs represent mean ± SE of three separate experiments. *p* value was determined by two‐tailed, paired *t* test. ∗, *p* < .001 vs. the levels of total **(A)** IgM or **(B)** IgG in the sera from untreated recipients. **(C, D):** Sera from allograft recipients and naïve BALB/c mice were harvested on postoperative day 8 (*n* = 8). FcγIII receptor and FcγII receptor on donor origin C57BL/6 (H‐2^b^) splenocytes were blocked with rat anti‐mouse CD16/CD32 and then treated with various dilutions of recipient serum. Donor specific **(C)** IgM or **(D)** IgG antibody binding was detected with fluorescently labeled goat F(ab’)_2_ anti‐mouse IgM (μ chain‐specific) or anti‐mouse IgG (H+L chain‐specific) and analyzed by flow cytometry. Graphs represent mean ± SE of three separate experiments. *p* value was determined by one‐way analysis of variance. ∗, *p* < .001 vs. the levels of donor specific **(C)** IgM or **(D)** IgG antibody in the sera from untreated recipients. Abbreviation: ERC, endometrial regenerative cell.

Results from ELISA revealed that ERCs significantly decreased total serum levels of IgM and IgG as compared with those in untreated recipients (*p* < .001 for IgM and IgG; Fig. 6A, 6B). Our next goal was to determine whether ERC‐mediated inhibition of antibody production was alloantigen specific. The levels of donor‐specific antibodies in the sera of ERC‐treated recipients were quantitated as compared with untreated transplant recipients and naïve mice. As shown in Figure 6C and 6D, donor‐specific IgM and IgG in the sera from untreated transplant recipients were remarkably high, as the levels of donor specific IgM and IgG in untreated recipients’ serum were titratable and decreased proportionally with increasing serum dilution. In contrast, donor‐specific IgM and IgG in the sera from ERC‐treated transplant recipients were significantly lower than those from untreated recipients (*p* < .001; Fig. 6C, 6D), which were, in fact, comparable with the control levels in naïve mice (baseline levels; Fig. 6C, 6D).

**Figure 7:**
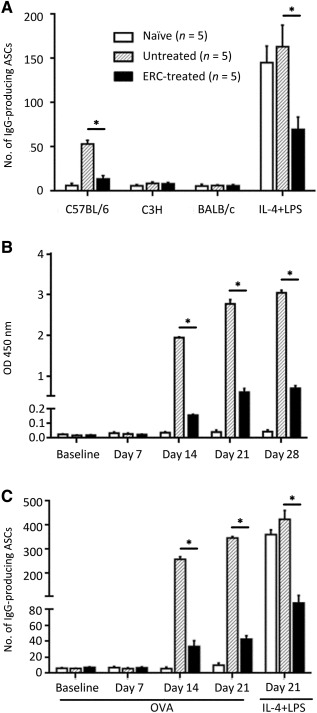
ERCs inhibit donor‐specific and total IgG ASCs in cardiac allograft recipients, as well as the levels of anti‐OVA antibody and the frequency of anti‐OVA antibody‐secreting B cells of OVA‐vaccinated mice. **(A)** To identify anti‐donor‐specific ASCs, splenic CD19^+^ B cells of allograft recipients (postoperative day 8) or naïve BALB/c mice were exposed to various cell lysates (donor C57BL/6, third‐party C3H, or syngeneic BALB/c) coated on ELISpot plates (*n* = 5). Uncoated wells supplemented with IL‐4 (100 U/ml) and LPS (2 μg/ml) served as positive controls to indicate the total IgG‐secreting cells within the splenic CD19^+^ B‐cell pool. **(B):** Anti‐OVA antibody within the circulation of OVA‐vaccinated mice. IgG anti‐OVA antibody titers within the various serum samples collected from mice immunized with ovalbumin‐aluminum hydroxide and naïve mice (days –1, 7, 14, 21, 28; *n* = 5) were determined by enzyme‐linked immunosorbent assay. **(C):** To identify anti‐OVA‐specific ASCs, splenic CD19^+^ B cells of OVA‐vaccinated, or naïve BALB/c, mice were harvested at days 0, 7, 14, and 21 and exposed to OVA‐coated ELISpot plates (*n* = 5). Uncoated wells supplemented with IL‐4 (100 U/ml) and LPS (2 μg/ml) served as positive controls to indicate the total IgG‐secreting cells within the splenic CD19^+^ B‐cell pool at day 21 postpriming with OVA. **(A–C):** Graphs represent mean ± SE of three separate experiments.∗, *p* < .001 determined by one‐way analysis of variance. Abbreviations: ASCs, antibody‐secreting cells; ERC, endometrial regenerative cell; LPS, lipopolysaccharide; OD, optical density; OVA, ovalbumin.

The effect of ERC treatment on the number of ASCs in cardiac allograft recipients was also examined using ELISpot assay. As shown in Figure 7A, antidonor ELISpot specificity was illustrated by significant numbers of ASCs detected only in the presence of donor (H‐2^b^) antigen. ERC treatment was capable of significantly inhibiting the frequency of antidonor ASCs and also was able to downregulate the total number of ASCs that could produce IgG (antigen‐negative wells supplemented with IL‐4 + LPS) compared with the untreated group (*p* < .001; Fig. 7A).

### ERCs Inhibit Anti‐OVA Antibody Levels and Reduce the Frequency of Anti‐OVA Antibody‐Secreting B Cells in OVA‐Vaccinated Mice

To study the effects of ERCs on the humoral responses in vivo, we attempted to immunize our allograft recipients with an OVA‐Alum. Unfortunately, vaccination induced accelerated cardiac allograft rejection in both the ERC‐treated and untreated allografts, such that all cardiac transplants had a MST of 3–4 days, which was similar to other transplant systems [38]. Thus, to study the effects of ERCs on the humoral response to a “non‐graft” antigen, nontransplanted mice were used instead and were vaccinated with the same OVA‐Alum suspension using a prime‐boost regimen on days 0 and 7.

As expected, high levels of anti‐OVA‐specific antibody (Fig. 7B), and a large number of anti‐OVA‐specific antibody secreting CD19^+^ B cells (Fig. 7C) in untreated mice, were observed upon OVA‐Alum immunization, which were significantly inhibited by ERC treatment (*p* < .001). Anti‐OVA specificity was illustrated only by the higher numbers of ASCs seen in the presence of OVA and not by those with the negative control keyhole limpet hemocyanin protein (data not shown). ERC treatment not only significantly inhibited the frequency of anti‐OVA ASCs but also decreased the total number of ASCs that could produce IgG (antigen‐negative wells supplemented with IL‐4 + LPS) compared with untreated (*p* < .001; Fig. 7C).

## Discussion

In a transplant setting, alloantibody binding to graft endothelial cells activates complement‐mediated destruction, facilitating significant graft injury [1]. ERCs share the similarities of MSCs in both phenotypic characteristics and the immunomodulatory properties [23, 24]. We have recently reported in an experimental colitis model that ERC‐treated mice exhibited significantly decreased MHC‐II expression on DCs, as well as reduced T‐cell population and activation [27]. To our knowledge, the present study for the first time shows the effect of ERCs on B‐cell responses both in vitro and in vivo. Although acute rejection is largely T‐cell mediated, the results of this work indicate that ERCs mediate B‐cell suppression as well, which could be at least part of the efficacy of ERC treatment against graft rejection.

ERCs inhibit LPS‐induced polyclonal B‐cell proliferation and antibody production in a dose‐dependent manner and do not have any cytotoxicity. This is consistent with previous observations showing that the inhibition of B‐cell proliferation is independent of apoptosis but a cell cycle arrest [30, 39]. Meanwhile, ERC‐treated B cells had significantly lower levels of cell surface CD80, CD83, and CD86 expression in response to stimulation with LPS. Because CD80 and CD86 trigger critical costimulatory signals to T cells when engaged with CD28 on the T‐cell surface, decreased expression of these markers on B cells suggests that ERC‐treated B cells may also have a reduced potential to stimulate T cells.

Indeed, in this study, we demonstrated that treatment of ERCs doubled the mean survival time of cardiac allografts, which was associated with much less intragraft antibody deposition. Meanwhile, total antibody as well as graft‐specific antibody levels in the sera of ERC‐treated recipients was markedly lower than those in untreated recipients, suggesting that ERC treatment suppresses de novo production of donor‐specific antibody in response to heart transplantation. In addition, regarding the inhibition of IgM production, which is not promoted by T cells [40], it appears that ERCs exert direct effects on B cells, in line with our previous finding [36]. This is supported by the observation that ERCs inhibited B‐cell function upon stimulation with TI antigen in the in vitro assays. However, controversies exist regarding the mechanism of MSCs on B cells [30, 40, 41]. The discrepancies may be explained by different sources of B cells and MSCs, as well as different stimulation methods [18]. The mechanism of ERCs in B‐cell suppression needs to be further investigated.

Because ERCs actually targeted B‐cell function in this study, the therapeutic use of ERCs in transplantation may be more effective than currently available therapies. Although antibody depletion strategies such as plasmapheresis or immunoabsorption can be effective in the removal of antibody, they remove *all* antibody and not only donor‐specific antibody, and they do little to prevent antibody production [42]. Because ERC therapy inhibits the production of alloantibody, it could potentially be used after depletion of circulating antibody at the time of transplantation to suppress de novo antibody production or the rapid production of antibody by a memory B‐cell response.

Because long‐lived plasma cells and memory B cells typically present in presensitized transplant recipients do not express the same markers as naïve B cells, therapeutic antibodies such as antithymocyte globulin, alemtuzumab, or anti‐CD20 monoclonal antibody (that all target resting B cells for depletion) do not affect mature B‐cell types [42]. By way of inhibiting antibody production, ERCs could potentially suppress the B‐cell immune response regardless of B‐cell differentiation state.

Moreover, although depletion of B cells from the immune system is immediately beneficial in terms of transplantation, it carries the risk of vulnerability of the host to various infections with pathogenic organisms and cancers caused by long‐term pan‐specific immunosuppression of B cells. In contrast to B‐cell depletion therapies, the observation that ERCs do not eliminate B cells from the circulation suggests the possibility of maintaining B‐cell responsiveness to nonallogenic antigens, which may allow the host to remain immunocompetent following ERC treatment. Whether the long‐term ERC therapy could result in a general effect on nonalloantibody must be further elucidation.

In the in vivo cell tracking by Hida et al., enhanced green fluorescent protein‐labeled menstrual blood‐derived mesenchymal cells could be observed on the host heart 2 weeks after cell transplantation, [26] suggesting that human ERCs were not rejected by the mouse recipients. Meanwhile, we previously found that human ERCs were able to suppress cell proliferation and proinflammatory cytokine production in a mouse‐mixed lymphocyte reaction [23]. Collectively, it appears that ERCs have no immunogenicity even if they are from humans in an immune competent xenogeneic animal. In our study, the effectiveness of ERCs was not mouse‐strain dependent, because similar graft prolongation was attained in a C3H‐to‐C57BL/6 transplant model (data not shown) as with the C57BL/6‐to‐BALB/c shown here. More in‐depth studies are warranted to clarify how ERCs are involved in the procedure.

## Conclusion

In addition to inactivation of T‐cell responses [27], this study demonstrates that ERCs inhibit B‐cell activation and differentiation with reduced antibody production in both in vivo models, cardiac allo‐transplantation and OVA‐Alum vaccine. There may be many possible mechanisms by which human ERCs inactivate B cells in these mouse hosts, such as indirectly from their T‐cell suppression and complement depletion. Our in vitro data clearly show that ERCs negatively regulate B‐cell maturation and activation directly without affecting their viability, suggesting that it will be possible that ERC‐mediated B‐cell suppression, at least in part, contributes to prolonged allograft survival in this preclinical model. With its unique features including ease of collection, relatively unlimited sources, immunomodulatory effects, and hypoimmunogenicity, as well as the lack of tumorigenesis or tumor acceleration, ERCs could become an attractive novel source of stem cells for cytotherapy for preventing and/or treating acute‐ and chronic‐humoral rejection following transplantation.

## Author Contributions

X.X. and X.L.: conception and design, collection and/or assembly of data, data analysis and interpretation, manuscript writing, final approval of manuscript; X.G. and P.S.: collection and/or assembly of data, provision of study material or patients, final approval of manuscript; B.Z., W.T., and H.H.: collection and/or assembly of data, data analysis and interpretation, final approval of manuscript; C.D.: data analysis and/or interpretation, final approval of manuscript; H.W.: conception and design, financial support, administrative support, manuscript writing, final approval of manuscript.

## Disclosure of Potential Conflicts of Interest

The authors indicated no potential conflicts of interest.
